# Open surgical treatment of unicameral bone cysts: A retrospective data analysis

**DOI:** 10.1007/s00508-023-02267-4

**Published:** 2023-08-31

**Authors:** Kevin Döring, Géraldine D. Sturz, Gerhard Hobusch, Stephan Puchner, Reinhard Windhager, Catharina Chiari

**Affiliations:** 1 Department of Orthopedics and Trauma Surgery, Medical University of Vienna, Vienna, Austria; 2 Department of Orthopedics and Trauma Surgery, Vienna General Hospital, Medical University of Vienna, Währinger Gürtel 18–20, 1090 Vienna, Austria

**Keywords:** Tumor-like lesion, Bone tumor, Surgical oncology, Curettage, Bone graft

## Abstract

**Background:**

A variety of treatment options for unicameral bone cysts (UBC) exist. The controversy of open management of UBC is discussed. The aim of this study was to analyze a single institution’s experience in the open surgical treatment of UBC.

**Patients and methods:**

By retrospective analysis of the Vienna Bone and Soft Tissue Tumor Registry, 119 patients with open surgery and histologically verified UBC with a mean follow up of 4.8 years (range 1–30 years) were included. Lesion treatment failure was defined as surgically addressed UBC undergoing revision surgery due to persistence or recurrence.

**Results:**

Local revision-free survival for lesion treatment failure was 93% after 1 year, 80% after 2 years, 60% after 5 years and 57% after 10 years. Of the patients 34 (29%) had at least 1 revision surgery due to lesion treatment failure. We found that patients with lesion treatment failure were younger (*p* = 0.03), had UBC with less minimal distance to the growth plate (*p* = 0.02) and more septation chambers in radiologic imaging (*p* = 0.02). Patients with open revision surgery were less likely to require a second revision due to lesion treatment failure than patients with percutaneous revision surgery (*p* = 0.03).

**Conclusion:**

Open surgery for UBC can only be recommended as reserve treatment in younger children with actively growing lesions. Open UBC surgery carries a relatively high risk of almost 30% of lesion treatment failure and therefore the indications should be limited to extensive osteolysis with high risk of pathological fractures, lesions with displaced pathological fractures, and lesions with an ambiguous radiological presentation that require tissue collection.

## Introduction

Surgical management of symptomatic unicameral bone cysts (UBC) warrants an ongoing discussion. As high intraosseous pressure by venous obstruction and enzymatic lysis are mostly considered responsible for UBC pathogenesis, modern approaches aim for lesion control and fracture prevention by cyst wall decompression with additional injections of sclerotherapeutic agents or cortisone [1–3]. In these operations, bone healing may be further promoted by bone marrow aspirate, demineralized bone matrix or other bone substitutes [4]. In recent years percutaneous techniques gained attention due to an acceptable risk of recurrence and low surgical site morbidity, which is especially important in a typically young patient collective. This shift to less invasive surgery even prompted some authors to proclaim that aggressive open procedures are unjustified in the surgical treatment of UBC [5–7]; however, percutaneous treatment success varies, and repeated interventions may still be necessary in a considerable percentage of patients due to recurrent or persistent UBC [1, 7–9]. Finding an indication for percutaneous surgery might be further complicated due to an inappropriate cyst size or morphology of fractured UBC. Thus, traditional curettage and bone grafting of symptomatic UBC may still be indicated in larger lesions with progressive growth and a high risk or already present pathological fractures with the need of additional internal fixation or tissue sampling, especially in weight-bearing bones. In these cases, open surgery represents a commonly used technique to fully mechanically remove the inner cyst layer and to ensure a dense defect packing [10]. In contrast to these potential benefits, prior studies reported mixed outcomes after curettage and bone grafting in terms of local lesion control rates and bone healing, with treatment failure rates ranging between 25% [1] and 64% [4, 11].

As long-term follow-up data regarding the open surgical treatment of UBC of larger case series are still sparse in the literature and as additional data are required to profile advantages and disadvantages of different UBC treatment modalities, we aimed to answer the following questions: (1) how frequent was the lesion treatment failure (LTF) rate after open surgical treatment of UBC? (2) Were there associations with patient parameters or treatment modalities that led to a higher rate of LTF?

## Patients and methods

### Patients

After acceptance of the research proposal by the Ethics Committee of the Medical University of Vienna, a retrospective data analysis of prospectively collected data in the patient administration system of the Medical University of Vienna identified 205 patients who received surgery of histologically verified unicameral bone cysts between 1988 and 2019. After exclusion of patients with (1) a clinical follow-up between surgery and last visit to the outpatient clinic of less than 1 year (*n* = 51), (2) missing surgical protocols (*n* = 17), (3) first surgery at our institution for recurrent UBC (*n* = 4) and (4) percutaneous treatment for primary UBC (*n* = 14), 119 patients were included for statistical analysis. Acquired data included basic demographic parameters, histopathological and radiological records, surgical documentation and follow-up records. The patient collective consisted of 79 male and 40 female patients with a mean (minimum–maximum) age of 13 years (5 months–47 years) at the time of surgery (Table 1).

**Table 1:** General characteristics and surgical parameters

Parameter	Patients (*n* = 119)	Lesion treatment failure (*n* = 34)	Lesion treatment success (*n* = 85)	*p*
Mean age at surgery	13 years (5 months–47 years)	10.5 years (5 months–40 years)	14.1 years (3–47 years)	**0.03**^b^
Mean follow-up time	4.8 years (1–30 years)	5.7 years (1–16 years)	4.5 years (1–30 years)	0.1^c^
Mean time to revision surgery^a^	25.4 months (5–84 months)	25.4 months (5–84 months)	–	–
Sex (male/female)	79 (66%)/40 (34%)	18 (53%)/16 (47%)	61 (72%)/24 (28%)	0.1^d^
*Localization*				
Humerus	68 (57%)	23 (68%)	45 (53%)	0.2^d^
Femur	26 (22%)	8 (24%)	18 (21%)	0.7^d^
Tibia	11 (9%)	1 (3%)	10 (12%)	0.2^d^
Calcaneus	5 (4%)	1 (3%)	4 (5%)	0.8^d^
Fibula	3 (3%)	0	3 (4%)	0.5^d^
Radius	2 (2%)	1 (3%)	1 (1%)	0.9^d^
Scapula	1 (1%)	0	1 (1%)	0.7^d^
Acetabulum	1 (1%)	0	1 (1%)	0.6^d^
Ischium	1 (1%)	0	1 (1%)	0.5^d^
Ilium	1 (1%)	0	1 (1%)	0.6^d^
*Finding*				
Pathological fracture	72 (61%)	25 (74%)	47 (55%)	0.2^d^
Pain	28 (24%)	5 (15%)	23 (27%)	0.4^d^
Incidental	12 (10%)	1 (3%)	11 (13%)	0.3^d^
N/A	7 (6%)	3 (9%)	4 (5%)	0.9^d^
*Primary surgical treatment*				
Open surgery and curettage	119 (100%)	34 (100%)	85 (100%)	–
Burring	79 (66%)	22 (65%)	57 (67%)	0.6^d^
Phenolization	40 (34%)	14 (41%)	26 (31%)	0.2^d^
Filling total	112 (94%)	32 (94%)	80 (94%)	0.7^d^
Autogenous bone filling	12 (10%)	3 (9%)	9 (11%)	0.4^d^
Allograft bone chips filling	96 (81%)	28 (82%)	68 (80%)	0.8^d^
Strut graft bone filling	7 (6%)	3 (9%)	4 (5%)	0.4^d^
Bone substitute material filling	15 (13%)	4 (12%)	11 (13%)	0.9^d^
Plate osteosynthesis	38 (32%)	9 (26%)	29 (34%)	0.5^d^
Decompression screw	1 (1%)	0	1 (1%)	0.5^d^
External fixator	1 (1%)	0	1 (1%)	0.6^d^

^a^Revision surgery for recurrent or persistent UBC

^b^Cox regression, bold = statistical significance

^c^T‑test for independent samples

^d^χ^2^-test

*N/A* Not available

### Surgical indications

Patients were usually referred to our outpatient clinic with pain in an extremity and X-rays showing a unicameral osteolysis of the long bones in the proximity of the growth plate without signs of malignancy. In cases of ambiguous presentation in X‑rays, especially with possible soft tissue involvement or expansion of the metaphyseal cortex over the width of the growth plate, additional magnetic resonance imaging (MRI) was usually undertaken to distinguish the lesion from possible differential diagnoses. Absolute surgical indications were typically found in patients with uncertain radiographic diagnoses and the need for a biopsy and in patients with displaced pathological fractures of weight-bearing bones. Relative indications for open surgical treatment of UBC were typically found in symptomatic patients with persistent pain or in growing lesions of weight-bearing bones to avoid pathological fractures. In cases of nondisplaced pathological fractures of non-weight-bearing bones at initial presentation, conservative treatment with immobilization was usually carried out for 4–6 weeks prior to surgery. Pathological fractures at initial presentation were very common as indications for surgery, with 72 of 119 patients (61%) showing pathological fractures in X‑rays at the first visit in the outpatient clinic (Table 1).

### Surgical technique

Surgery was performed under fluoroscopic guidance by exposure of the affected bone area and access to the bone cyst by preparation of a cortical bone window or burring. The lesion lining was then removed by manual curettage using sharp, straight and angulated curettes and sent for histological examination. In many patients, additional mechanical burring with rose head burrs and cavity phenolization with phenol-soaked swabs was performed to eradicate potentially remaining septa and membranes. The cavity was thereafter filled with autogenous or allogenous bone chips, strut grafts in cases where additional stability was required or bone substitute material. The bone was collected from the iliac crest in cases of autogenous bone grafting. In cases of unstable pathological fractures, additional plate osteosynthesis was performed.

### Follow-up examination

Patients were usually invited for a follow-up examination 2 weeks, 6 weeks, 4–6 months, 12 months and annually thereafter after surgery. Mean follow-up time was 4.8 years (range 1–30 years). Follow-up examination was performed in our outpatient clinic by surgical site inspection and X-ray imaging. At least 1 year of radiographic follow-up was available in 93 of 119 patients (78%), and a complete radiological assessment, including preoperative, intraoperative, and postoperative X‑ray imaging as well as X‑rays 6 months and 1 year after surgery and at last follow-up was available in 59 of 119 patients (50%). At the time points after surgery, modified Neer scores were assessed. In preoperative X‑ray imaging, the UBC volume as well as the distance of the UBC to the nearest growth plate was measured. Additionally, UBCs were grouped by cyst septation and the number of visible septation chambers. In cases of suspected recurrences or persistent cysts, MRI was performed for lesion size quantification and surgery planning.

### Modified Neer classification

Local recurrence and persistence were defined according to the modified Neer classification [12]. The modified Neer classification divides the postoperative radiologic findings into four groups. Grade I (“healed”) describes a complete or almost complete filling of the initial lesion, with or without small radiolucent areas under 1 cm in size. Grade II (“healing with defect”) describes radiolucent areas under 50% of the bone diameter, and with enough cortical thickness to prevent fracture. Modified Neer classification grade III (persistent cyst) specifies postoperative results that show radiolucent areas over 50% of the bone diameter, with a thin cortical rim and a risk of fracture. Grade IV (recurrent cyst) applies to UBCs reappearing in a previously obliterated area or a residual radiolucent area that increases in size.

### Definition of lesion treatment failure

Cases of LTF were defined as UBCs that were revised in a second surgery either due to persistence or local recurrence after initial surgery, corresponding to modified Neer classification grade III (persistence) or grade IV (recurrence). Persistent cysts were defined as surgically addressed UBC with graft resorption or persistent radiolucent areas greater than 50% of the initial lesion at risk for fracture, while recurrent cysts were defined as UBC after surgery with reappearance in a previously obliterated area or a residual radiolucent area that has increased in size.

### Statistical analysis

Descriptive statistics were used to detect frequencies, means, medians and ranges. Differences between means were tested by independent *t*-test for continuous variables. Differences between frequencies were tested by χ^2^-tests. Differences in frequencies of revision after initial surgery and after revision surgery were tested by a binomial test. Frequencies of LTF regarding the decade in which the surgery was performed were analyzed using a χ^2^-test, and total frequencies of surgeries per decade were analyzed using a binomial test. Survival analyses were performed using Kaplan-Meier plots. Potential influencing factors on LTF were analyzed using a Cox regression model. Statistical significance was determined by a *p*-value < 0.05. Statistics were performed using IBM SPSS Statistics 26 (IBM, Armonk, NY, USA) software.

## Results

### Lesion treatment failure rate after open surgical UBC treatment

**Fig. 1:**
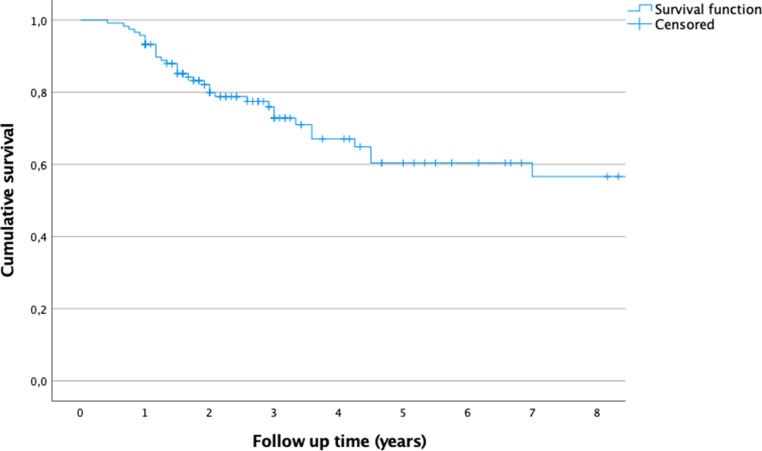
Graph showing the local revision-free cumulative survival of treatment failure over the follow-up time. Local revision-free survival of treatment failure was 93% after 1 year, 80% after 2 years, 60% after 5 years and 57% after 10 years

**Fig. 2:**
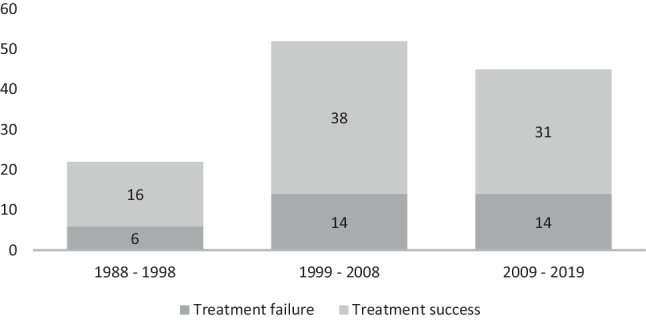
Total open UBC surgery absolute frequencies with respect to the different decades in which the surgery was performed

Local recurrence was reported in 30 patients (25%), while 8 patients (7%) had persistent cysts after treatment. Of these patients 34 (29%) had at least 1 revision surgery due to UBC recurrence or persistence and were thus considered as LTF, 10 UBCs (8%) required 2 revisions to achieve bone healing. Local revision-free survival of LTF was 93% after 1 year, 80% after 2 years, 60% after 5 years and 57% after 10 years (Figure 1). The mean time from initial surgery to first revision due to LTF was 25 months (range 5‑84 months). UBCs after revision surgery were not more likely to require revision surgery due to LTF than initial UBC (10/34 versus 34/119, *p* = 0.5). Patients with open revision surgery were less likely to require second surgery for LTF than patients with percutaneous revision surgery (*p* = 0.03, Table 2). To further analyze different time periods of surgery, the frequencies of surgery were divided into decades from 1988–1998, 1999–2008 and 2009–2019. Although no difference regarding LTF frequencies were found, there was an increase in open UBC surgery performed in the latter periods in comparison to the first period from 1988–1998 (*p* = 0.001, Fig. 2).

**Table 2:** Lesion treatment Failure and revision analysis

Parameter		Lesion treatment failure (*n* = 34)	Revision lesion treatment failure (*n* = 10)	*p*
*Recurrences/persistent cysts*		30/8	–	–
Recurrences/persistent cysts without revision surgery		4/0	–	–
Open revision surgery		23 (68%)	4	**0.03**^b^
Percutaneous revision surgery		11 (32%)	6	**0.03**^b^
Number of 3rd revisions for treatment failures		0	–	–
*Total number of revision surgeries*^a^				
Patient with one revision surgery		38	–	–
Patients with two revision surgeries		22	–	–
Patients with three revision surgeries		2	–	–

^a^Including all reasons for revision surgery (recurrence, persistent cysts, material removal surgery, correction osteotomy)

^b^χ^2^-test, bold = statistical significance

### Complications and consecutive procedures

Regarding postoperative complications, one patient with a pathological fracture of a proximal humeral UBC at initial presentation sustained a fracture dislocation after surgery which required repositioning under sedoanalgesia, whereas two patients fractured the proximal humerus after surgery due to a fall. One of these patients required open reduction and internal fixation of the humerus. Patients with a fracture after surgery did not suffer from LTF before or after fracture treatment. Wound infections occurred in two patients, whereby one patient needed revision surgery with debridement and filling material removal due to the infection. Intermittent radial nerve palsy was described in three patients, with one patient requiring radial nerve neurolysis. Regarding sequelae in the postoperative treatment course, one patient developed coxa vara and underwent osteotomy of the proximal femur, while one additional patient sustained avascular necrosis of the femoral head that required femoral varus osteotomy and Salter pelvic osteotomy. One patient presented with a significant humeral growth deficiency and underwent a humeral osteotomy with insertion of a lengthening nail 8 years after initial surgery.

### Parameters associated with lesion treatment failure

**Fig. 3:**
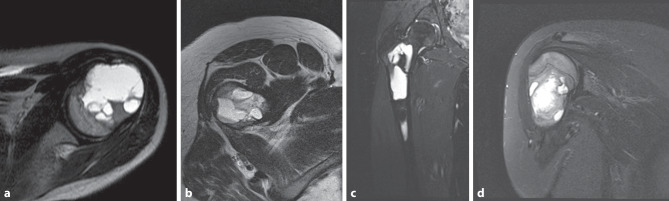
Examples of multichambered UBC in preoperative MRI. **a** Axial MRI-image of a proximal humeral UBC, **b** axial MRI-image of a proximal femoral UBC, **c** frontal MRI-image of a proximal femoral UBC, **d** frontal MRI-image of a proximal humeral UBC

After controlling for possible confounding variables, such as patient sex or treatment modalities, such as additional cavity burring, phenolization or osteosynthesis, we found that patients with LTF were younger than patients with treatment success (10.5 versus 14.1 years, *p* = 0.03, Table 1). Additionally, patients with LTF presented with UBC with less distance to the growth plate in preoperative X‑rays (*p* = 0.02) and more septation chambers in radiologic imaging (*p* = 0.02, Table 3, Fig. 3). No further parameters, especially regarding surgical modalities, such as cavity burring using a high-speed burr, additional phenolization, the type of bone graft used for reconstruction, additional osteosynthesis, or cyst localizations showed an influence on revision-free survival.

**Table 3:** Preoperative and postoperative radiological parameters

Parameter	Patients (*n* = 59)	Lesion treatment failure (*n* = 20)	Lesion treatment success (*n* = 39)	*p*
Lesion volume	55.4 (0.7–234.4) cm^3^	54.9 (0.7–165) cm^3^	55.7 (1.6–234.4) cm^3^	0.96^a^
Distance UBC to nearest growth plate	34.4 (0–112) mm	21 (0–80) mm	44.5 (0–112) mm	**0.02**^a^
Cyst septation (yes/no)	24 (41%)/35 (59%)	12 (60%)/8 (40%)	12 (31%)/27 (69%)	**0.03**^b^
Number of visible chambers	1.6 (1–5)	2 (1–5)	1.4 (1–3)	**0.02**^a^
Modified Neer score 6 months	1.6 (1–4)	–	–	–
Modified Neer score 12 months	1.9 (1–4)	–	–	–
Modified Neer score 2 years	1.5 (1–4)	–	–	–
Modified Neer score at last follow-up	1.9 (1–4)	–	–	–

Only patients with a full set of radiological parameters (*n* = 59) were included for statistical analysis. Data displayed as mean (standard deviation) in continuous variables.

^a^T‑test for independent samples

^b^χ^2^-test

## Discussion

**Fig. 4:**
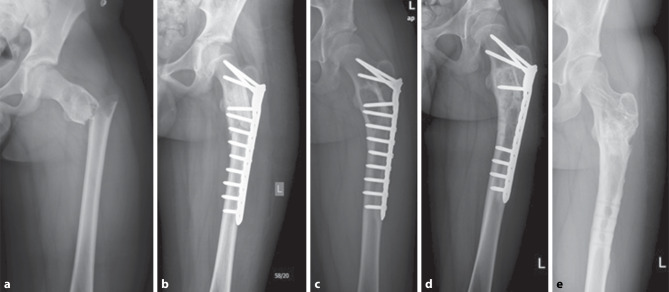
A 12-year-old patient with subtrochanteric pathologic fracture due to an UBC before (**a**) and after (**b**) open surgery with curettage, burring, filling with a strut graft and allograft bone chips, and plate osteosynthesis. In follow-up X‑rays 18 months after surgery, recurrence in the trochanteric region was visible (**c**), which led to open revision surgery with screw removal, curettage, phenolization, and filling with bone substitute chips. (**d**) The bone fixation was removed 3 years after recurrence (**e**)

**Fig. 5:**
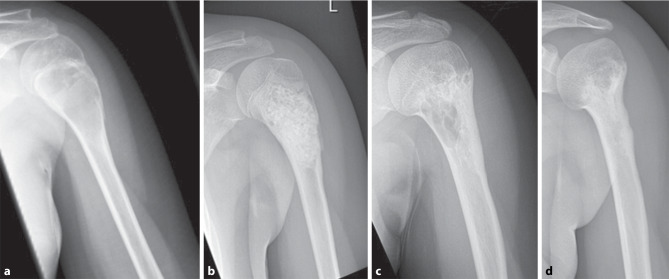
A 10-year-old patient with pain and a proximal humerus osteolysis in X‑rays (**a**) which led to open curettage, burring, phenolization and filling with allograft bone chips (**b**). A recurrent osteolysis in the proximal humerus was visible (**c**) 3 years after surgery for UBC, and additional curettage, burring and cavity filling with allograft bone chips was performed. No further recurrence was observed 2 years after revision surgery (**d**)

There is high variety and thus ongoing discussion regarding surgical UBC treatment. Additional data are required to further contour advantages and disadvantages of modern treatment choices [13]. This study was performed to evaluate long-term follow-up revision-free survival outcomes of open surgical UBC treatment of a single institution. We found 29% (34/119) of patients with need of revision after initial surgery due to recurrent or persistent UBC (Fig. 4). Additional surgical modalities, like phenolization or cavity burring, did not alter the survival of LTF of patients in this study (Fig. 5). A young patient age and proximity to open growth plates were linked to LTF. Information found in this study should help treating surgeons in finding indications for open UBC surgery and raise awareness in postoperative care of patients with a higher likelihood of recurrent or persistent UBC after initial treatment.

### Limitations

As a retrospective data analysis, this study comes with several limitations.

Documentation of physical limitations and functional outcomes was not standardized in outpatient clinic protocols, and thus was not analyzed in this study. This is an important limitation in an ongoing discussion between open and percutaneous surgery in UBC treatment, as functional impairments and surgical site morbidity may be main advantages of a less invasive treatment; however, because of this limitation, conclusions of this study are solely limited to surgical outcomes, and further studies analyzing functional results and rehabilitation protocols of different treatment strategies are needed for further comparison.

With an inclusion period of over 30 years diagnostic and treatment modalities, surgical indications and surgeons changed and developed. A long inclusion period was deemed necessary to achieve a solid patient number for statistical analysis, and a review of surgical protocols showed that general approaches to open UBC surgery did not significantly change in the inclusion period. Our analysis of the different decades of surgery supports this comparability, as no differences between the frequencies of LTF between the decades were found. An increase of open UBC surgery in the recent decades might be partially explained by an improvement of diagnostic possibilities; however, there are numerous possible confounding variables that could have also influenced the total numbers of open UBC surgeries performed in one specific decade, such as surgeons’ experience to indicate this treatment form, or patient reluctance to undergo surgery.

Due to the long inclusion period, missing data of radiologic imaging need to be reported, which led to an inability to assess radiologic images of early period UBC. Only surgical and general patient parameters of these patients were available.

### Treatment success in comparison to literature

A LTF rate of 29% (34/119) was found after open curettage. This result is supported by literature, with LTF rates after curettage ranging between 25% and 64%, and is generally comparable to minimally invasive UBC treatment, with failure rates after first treatment ranging between 0% and 78% [4, 9, 11, 12, 14]. As a potential self-limiting disease, UBC treatment invasiveness should be carefully balanced with respect to recurrence and pathological fracture. A personalized approach to surgery should be tailored to every patient due to high heterogeneity in cyst appearance, localization, and growth. Based on the success of modern percutaneous treatment options, conventional open surgery should foremostly be reserved for actively growing UBC of weight-bearing bones with a high fracture risk or in UBC with an ambiguous presentation in radiologic imaging and the need to collect tissue biopsies [7]. When indicated, open curettage, bone grafting and potential bone fixation come with an acceptable risk of recurrence.

Although an inherent worse local recurrence rate may be suspected in recurrent or persistent UBC requiring revision treatment, this was not the case in this study, as UBC revisions for LTF showed a comparable rate to UBC undergoing primary surgery. These results might be partially attributed to a higher patient age at second surgery and possibly concomitant less expansive UBC properties, as well as a possibly more aggressive surgical curettage in patients with closing growth plates [11]. Additionally, acceptable re-revision rates may be credited to an aggressive regimen of indicating surgical revisions in postoperative recurrences, as revisions were usually performed early in small but growing lesions.

**Fig. 6:**
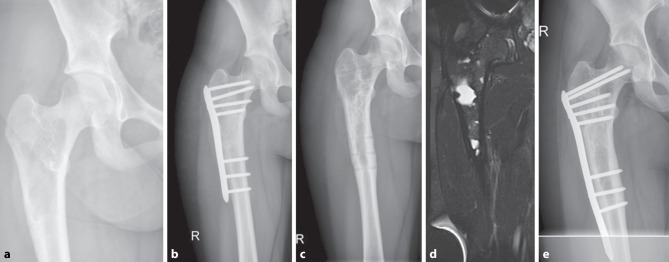
A 12-year-old patient with a proximal femoral UBC (**a**) received curettage, burring, filling with allograft bone chips and plate fixation in surgery. (**b**) Material removal surgery was performed 20 months afterwards (**c**) and  5 years later a progressive recurrent osteolysis was observed in follow-up MRI (**d**), which led to revision curettage, burring, filling with allograft bone chips and plate fixation (**e**)

In revision cases, open surgical treatment showed superior surgical outcomes in comparison to percutaneous surgery in this study. In comparison to large unicameral lesions in primary UBC, UBC recurrences may show morphologic changes after initial open surgery with dense cavity packing, as multiple and smaller cyst lacunae with separating bone bridges may be observed in patients with persistent or recurrent lesions (Figure 6). This lesion fragmentation might impose additional complexity to percutaneously decompress and fill the whole bone cyst circumference. With open curettage and filling, possible smaller and septated residua might be accessed, and thus open revision surgery may be especially recommended in complex revision cyst morphologies after initial open treatment.

### Open UBC surgery in patients at risk for recurrence

Patient and lesion factors associated with LTF were younger patient age, less distance from the UBC to the growth plate, and more septation chambers in preoperative imaging. These results conform to existing literature, with an established distinction between active and latent UBC based on lesion distance to the growth plate [6, 15]. The exact distance to the growth plate for classifying an UBC as active remains imprecise between studies. Haidar et al. demonstrated a 2 cm distance as a positive predictor for the treatment outcome, while other studies suggest either less space or direct contact with the growth plate as distinction [3, 13, 16]. In our study, UBC with LTF had a mean distance to the nearest growth plate of 2.1 cm, but LTFs were reported up to a distance of 8 cm. Due to these results, we concur with Mascard et al. to base UBC activity levels, and thus potential fracture risk, on multiple radiologic and patient factors, such as cavity enlargement over time, multicameral lesion presentation, pain and patient age [17]. As the fracture risk is a main indication for surgery in an otherwise potentially self-limiting disease, treating surgeons need to be aware of these factors to guide their clinical decision making [3, 4].

## Conclusion

Open surgery for UBC can only be recommended as reserve treatment in younger children with actively growing lesions. Open UBC surgery carries a relatively high risk of almost 30% of lesion treatment failure and therefore indications should be limited to extensive osteolysis with high risk of pathological fractures, lesions with displaced pathological fractures, and lesions with an ambiguous radiologic presentation that require tissue collection.
